# IGF2 knockout reduces but does not abolish osteosarcoma growth *in vitro* and *in vivo*

**DOI:** 10.1002/2211-5463.70341

**Published:** 2026-09-12

**Authors:** Shun Yao, Marco Archetti

**Affiliations:** ^1^ Department of Biology Pennsylvania State University USA; ^2^ Huck Institutes of the Life Sciences Pennsylvania State University USA; ^3^ Present address: Department of Pharmacology and Cancer Biology Duke University USA

**Keywords:** cell proliferation, CRISPR‐Cas9, growth factor signaling, IGF2, osteosarcoma, xenograft

## Abstract

Insulin‐like growth factor 2 (IGF2) is implicated in osteosarcoma, but direct functional evidence of its role is lacking. Here, we used CRISPR‐Cas9 to knock out IGF2 in Saos2 osteosarcoma cells. Knockout cells showed reduced proliferation *in vitro* which was fully restored by exogenous IGF2. *In vivo*, knockout xenografts reached only ~25% of wild‐type tumor volume. Furthermore, transcriptomic analysis revealed depletion of angiogenesis‐related genes but no major changes in IGF2 pathway components. Finally, while integration with receptor dependency data suggested alternative signaling pathways, IGF2 loss remained functionally limiting. These findings demonstrate that IGF2 promotes osteosarcoma proliferation and tumor growth but is not strictly essential for survival.

## Graphical abstract

To test whether endogenous IGF2 promotes osteosarcoma growth, IGF2 was knocked out in Saos2 cells via CRISPR‐Cas9. KO cells showed reduced proliferation *in vitro*, and knockout xenografts in mice reached only ~25% of wild‐type tumor volume.

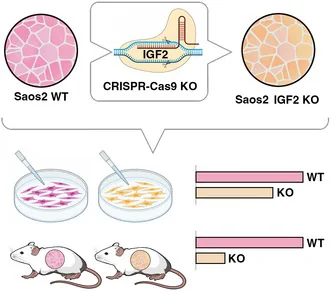

**Abbreviations**
ALK: Anaplastic lymphoma kinase
ATCC: American Type Culture Collection
AXL: AXL receptor tyrosine kinase
BMP: Bone morphogenetic protein
BMPR1A: BMP receptor type 1A
BMPR2: BMP receptor type 2
bp: Base pair(s)
Cas9: CRISPR‐associated protein 9
CCLE: Cancer Cell Line Encyclopedia
CLCF1: Cardiotrophin‐like cytokine factor 1
CO_2_
: Carbon dioxide
CRISPR: Clustered Regularly Interspaced Short Palindromic Repeats
DepMap: Cancer Dependency Map
DMEM: Dulbecco's modified Eagle medium
DNA: Deoxyribonucleic acid
ECM: Extracellular matrix
ERK: Extracellular signal‐regulated kinase
FBS: Fetal bovine serum
FDR: False discovery rate
FGF1: Fibroblast growth factor 1
FGF2: Fibroblast growth factor 2
FN1: Fibronectin 1
GAS6: Growth arrest‐specific 6
GOA: Gene Ontology Annotation
GSEA: Gene Set Enrichment Analysis
IACUC: Institutional Animal Care and Use Committee
IGF: Insulin‐like growth factor
IGF1R: Insulin‐like growth factor 1 receptor
IGF2: Insulin‐like growth factor 2
IGF2R: Insulin‐like growth factor 2 receptor
IL6ST: Interleukin 6 Signal Transducer
ITGAV: Integrin subunit alpha V
JAK: Janus kinase
kb: Kilobase(s)
KO: Knockout
LAMC1: Laminin subunit gamma 1
LIF: Leukemia inhibitory factor
LIFR: Leukemia inhibitory factor receptor
MAPK: Mitogen‐activated protein kinase
MDK: Midkine
MEK: MAPK/ERK kinase
MMP2: Matrix metallopeptidase 2
MSigDB: Molecular signatures database
mTOR: Mechanistic (mammalian) target of rapamycin
NES: Normalized enrichment score
NOD: Nonobese diabetic
NSG: NOD.Cg‐Prkdc^scid Il2rg^tm1Wjl/SzJ
OS: Osteosarcoma
PCR: Polymerase chain reaction
PDGFB: Platelet‐derived growth factor B
PDGFC: Platelet‐derived growth factor C
PDGFRA: Platelet‐derived growth factor receptor alpha
PI3K: Phosphoinositide 3‐kinase
PLAUR: Plasminogen activator, urokinase receptor
PTN: Pleiotrophin
RAF: Rapidly accelerated fibrosarcoma
RAS: Rat sarcoma
RNA: Ribonucleic acid
RTK: Receptor tyrosine kinase
SCID: Severe combined immunodeficiency
sgRNA: Single guide RNA
SL: Subcellular location
SPP1: Secreted phosphoprotein 1
ssGSEA: Single‐sample gene set enrichment analysis
STAR: Spliced transcripts alignment to a reference
STAT: Signal transducer and activator of transcription
TAE buffer: Tris‐acetate‐EDTA buffer
TGFB1: Transforming growth factor beta 1
TGFBR1: Transforming growth factor beta receptor 1
TGFβ: Transforming growth factor beta 1
THBS1: Thrombospondin 1
THY1: Thy‐1 cell surface antigen
TMM: Trimmed mean of M‐values
TNC: Tenascin C
TPM: Transcripts per million
UMI: Unique molecular identifier
V: Volts
w/v: Weight/volume
WT: Wild‐type

Osteosarcoma (OS) is the most common primary malignant bone tumor in children and adolescents and is associated with a substantial risk of recurrence and chemoresistance despite aggressive multimodal therapy [1, 2, 3]. One proposed mechanism of therapeutic resistance in many cancers involves the insulin‐like growth factor (IGF) axis, particularly insulin‐like growth factor 2 (IGF2) and insulin‐like growth factor receptors (IGF1R and IGF2R) [4, 5, 6, 7, 8, 9]. IGF2 binds IGF1R and IGF2 and regulates downstream signaling pathways [10, 11, 12], including PI3K/AKT, MAPK, and mTOR [13, 14, 15], which promote cell survival, proliferation, and growth. In OS [16], mutations of IGF signaling genes [17], variation in IGF1R copy number [18] and increased expression [19] are biomarkers of tumor progression. IGF2 also protects OS cells against chemotherapy [20]. Hence, targeting the IGF axis has emerged as a promising therapeutic target in osteosarcoma [19, 21, 22, 23, 24, 25]. Direct evidence of the effect of removing IGF2, however, is limited.

Osteoblast‐specific knockout of IGF1R in mouse reveals an essential role of IGF signaling in bone matrix mineralization [26]; knockdown of IGF2 decreases cell survival in colorectal cancer [27] and disrupts mitochondrial functions in the liver [28]. However, the role of endogenous IGF2 in osteosarcoma remains incompletely understood. Most mechanistic studies rely on exogenous IGF2 stimulation of wild‐type cells [16, 17, 18, 19, 20], leaving the relative contributions of autocrine versus paracrine IGF2 signaling [29, 30] unclear. CRISPR–Cas9 loss‐of‐function screens, such as those used in DepMap [31, 32] measure cell‐autonomous genetic dependencies, but are poorly suited to detecting dependencies on secreted proteins like growth factors, which can be supplied in trans by neighboring cells within the mixed populations used in pooled CRISPR assays. Clarifying the relative contributions of autocrine and paracrine IGF2 in OS survival could provide novel insights into potential therapeutic strategies targeting the IGF axis.

In the present study, we aimed to clarify the contribution of autocrine/cell‐intrinsic IGF2 to osteosarcoma growth—a question not resolved by prior exogenous‐stimulation studies or by pooled dependency screens, which are insensitive to secreted factors—using IGF2‐knockout Saos2 cells [33], osteosarcoma cells that retain osteoblast‐like characteristics and serve as a widely used model for bone‐forming cancer. We subjected Saos2 osteosarcoma cells to CRISPR‐Cas9–mediated knockout of IGF2 and assessed proliferation *in vitro* and *in vivo* using xenograft models. We performed RNA sequencing and ligand–receptor dependency analyses to explore potential compensatory pathways.

## Materials and methods

### Cell lines and culture

Human osteosarcoma Saos2 cells were obtained from the American Type Culture Collection (ATCC, HTB‐85) and were maintained in Dulbecco's Modified Eagle Medium (DMEM) supplemented with 10% fetal bovine serum (FBS) and 1% penicillin/streptomycin at 37 °C in a humidified incubator with 5% CO_2_.

### CRISPR‐Cas9 knockout of IGF2

**Fig. 1:**
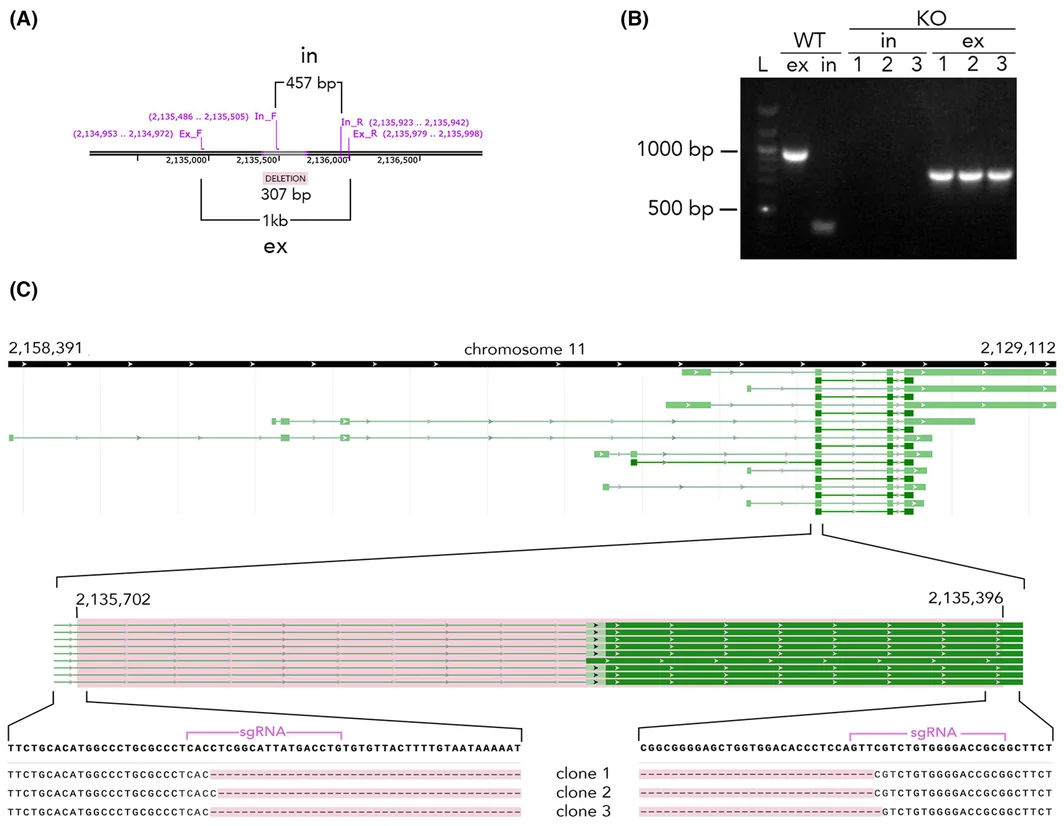
Knockout strategy and validation. (A) Wild‐type (WT) and knockout (KO; clones 1–3) templates were amplified using external ('ex') and internal ('in') primer combinations to generate diagnostic amplicons. Primer binding sites and expected product sizes are indicated. The internal primer pair is designed to yield an amplicon exclusively from the WT template, with no product generated from KO clones. (B) PCR products were resolved on a 1.5% agarose gel. Lane L: DNA ladder; Lanes WT: wild‐type cells; Lanes KO: knockout cell lines 1–3. Observed band sizes match expected amplicon sizes, confirming successful KO. (C) Genome browser‐style schematic of the NCBI RefSeq annotation of *IGF2* showing exon–intron organization, alternatively spliced transcripts, and predicted protein isoforms. Exons are represented by thick boxes and introns by thin connecting lines. An expanded view of the first common exon highlights the deletion (pink) and the sgRNA target sites used for CRISPR–Cas9‐mediated knockout. The deletion start and end positions for clone 1 are marked. Sanger sequencing alignments from the three KO clones are shown.

CRISPR‐Cas9 was used to generate IGF2 knockout (KO) cells. sgRNA off‐target effects were predicted using CHOPCHOP [34]. Two single guide RNAs targeting IGF2 at two positions on chromosome 11 (CGCGGTCCCCACAGACGAAC, at 2135379‐2135398; and CAGGTCATAATGCCGAGGTG at 2135686‐2135705) (Fig. 1) were delivered as ribonucleoprotein complexes via nucleofection using the Lonza 4D‐Nucleofector system. Transfections were performed following the manufacturer's recommended program for Saos‐2 cells (program DS‐150) and using the provided nucleofection solution. A pooled KO population was initially established, followed by single‐cell cloning to isolate individual knockout clones. To verify the knockout cell lines, genomic DNA was amplified via PCR using two distinct primer sets. The first set comprised an external primer pair flanking the deletion region (forward: 5′‐GATCATTCACCGAACGCACG‐3′, reverse: 5′‐ATGCACATGCTCTGTAGGGG‐3′), yielding a ~1 kb amplicon for wild‐type (WT) cells and a 739 bp amplicon for knockout (KO) cells. The second set consisted of an internal primer pair (forward: 5′‐AGAAGCACCAGCATCGACTT‐3′, reverse: 5′‐TGAGAAGTGGCGATGTGACG‐3′), with one primer situated within the deleted sequence to generate a 457 bp amplicon exclusively from WT cells (Fig. 1A). PCR products were subsequently analyzed by agarose gel electrophoresis: 2 μL aliquots of each PCR mixture were blended with 6× loading dye and resolved on a 1.5% (w/v) agarose gel prepared in 1× TAE buffer containing SYBR Safe nucleic acid stain. Electrophoresis was conducted at 90 V for 60 min, and a 100 bp DNA ladder was loaded concurrently as a molecular weight marker. DNA fragments were visualized under blue‐light illumination and documented using an Azure 200 gel imaging system (Fig. 1B). Successful deletion of IGF2 was further confirmed by Sanger sequencing (Fig. 1C).

### *In vitro* proliferation assays

Wild‐type (WT) and IGF2 knockout (KO) cells were seeded at equal densities in triplicate and grown under identical conditions. To test the effect of exogenous stimulation, various FBS concentrations or recombinant human IGF2 (R&D Systems #292‐G2) were added. Cells were harvested simultaneously when at least one population became confluent (approximately 5 days). Cell suspensions were mixed 1:1 with 0.4% trypan blue, and viable (trypan blue–excluding) cells were counted manually using a hemocytometer under brightfield microscopy. For each sample, 4 of the grid squares were counted, and the average was used to calculate cell density. Counts were performed by one blinded observer in triplicate biological replicates.

### *In vivo* xenograft studies

NOD.Cg‐Prkdc^scid Il2rg^tm1Wjl^/SzJ (NSG) mice (6–8 weeks old, female) obtained from The Jackson Laboratory (stock #005557) and maintained under pathogen‐free conditions in ventilated cages with nesting material in a light/dark cycle at 22 ± 2 °C and 40–60% humidity, with *ad libitum* access to standard irradiated rodent chow and filtered water. Mice were acclimatized for 1 week and allocated to WT or IGF2 KO groups (*n* = 6 per group) at random. WT or IGF2 KO Saos‐2 cells (5 × 10^6^ cells in 100 μL PBS) were injected subcutaneously into the right flank. Tumor volume, the primary outcome measure, was calculated as (length × width^2^)/2 from caliper measurements taken every 3–4 days. All animal procedures were conducted in accordance with the guidelines of the Institutional Animal Care and Use Committee (IACUC) of the Pennsylvania State University; the protocol was approved under permit number PROTO201901141. Humane endpoints were predefined and applied to all animals. Euthanasia was performed promptly when any of the following criteria were met: tumor size reaching an average diameter of 1.5 cm or a tumor volume exceeding 10% of body weight; body weight loss ≥20% compared to pretreatment baseline; body condition score <2; labored respiration; tumors interfering with normal locomotion; inability to access food or water; signs of dehydration; tumor ulceration with serous or bloody exudate; or tumor necrosis with exposure of internal tumor tissue. Once animals reached endpoint criteria, euthanasia was performed within the same day of assessment by gradual‐fill CO_2_ exposure (30–70% chamber volume displaced per minute) using a compressed gas cylinder with a calibrated flow regulator; death was confirmed by cessation of respiration and heartbeat, followed by cervical dislocation performed by trained personnel. No animals died before meeting criteria for euthanasia.

### Identification of growth factor–receptor pairs

Proteins annotated as growth factors (GOA: ‘growth factor activity [8083]’) and secreted mitogenic factors (GOA: ‘positive regulation of cell population proliferation [8284]’ or GOA: ‘negative regulation of apoptotic process [43066]’) with subcellular localization ‘Secreted’ (SL‐0243), excluding membrane proteins (SL‐0162) were identified in UniProt. Ligand–receptor pairs were obtained from the CellPhoneDB database (https://www.cellphonedb.org) [35]. Only simple one‐to‐one ligand–receptor pairs were considered; complex multimeric interactions were excluded.

### Gene expression and dependency analysis

Gene expression for candidate growth factors was obtained from the CCLE dataset in DepMap [31, 32] (release 23Q4) and reported as log_2_(TPM + 1). Gene dependency was quantified using DepMap probability of dependency values, where higher scores indicate a higher likelihood of essentiality in a given cell line.

### RNA sequencing and differential gene expression analysis

One WT and one IGF2‐KO Saos2 sample were submitted to Plasmidsaurus (Eugene, OR, USA) for 3′‐end RNA sequencing on an Illumina platform (single‐end, strand‐specific reads). Reads were filtered and trimmed using FastP v0.24.0, aligned to the human reference genome using STAR v2.7, and deduplicated using unique molecular identifiers (UMIs) with UMICollapse v1.1.0. Gene‐level counts were generated using featureCounts (subread v2.1.1) and normalized using the trimmed mean of M‐values (TMM) method. Functional enrichment was performed using single‐sample Gene Set Enrichment Analysis (ssGSEA) [36, 37] implemented via GSEApy v0.12 [38], against the MSigDB Hallmark gene set collection [39, 40]. As RNA sequencing was performed on a single sample per genotype, ssGSEA enrichment scores are reported descriptively; conventional phenotype‐permutation NES/FDR statistics, which require multiple replicates per group, could not be calculated. Quality control metrics, fold change values, and enrichment results are provided in the Data S1– S5.

## Results

### CRISPR‐Cas9‐mediated IGF2 knockout reduces osteosarcoma growth

To investigate the role of IGF2 in Saos2 cells, we designed single guide RNAs (sgRNAs) for CRISPR‐Cas9‐mediated knockout. Following single‐clone selection, we obtained three homozygous IGF2 knockout (KO) cell lines carrying a ∼306 bp deletion including the first common exon of all IGF2 isoforms annotated in the Cancer Cell Line Encyclopedia (CCLE) [41, 42]. In all annotated isoforms except one, which is not expressed in Saos2 cells according to CCLE data, this exon is also the first translated exon. Correct genomic editing was confirmed by PCR and Sanger sequencing (Fig. 1). The two sgRNAs have no predicted off‐targets with fewer than 3 mismatches and two potential off‐targets with three mismatches at chr4:75892340 and chr6:148379242, in introns of *PPEF2* and *SASH1*, respectively. Neither gene showed evidence of dependency in Saos‐2 cells according to DepMap Chronos scores (−0.09 and −0.04, respectively).

**Fig. 2:**
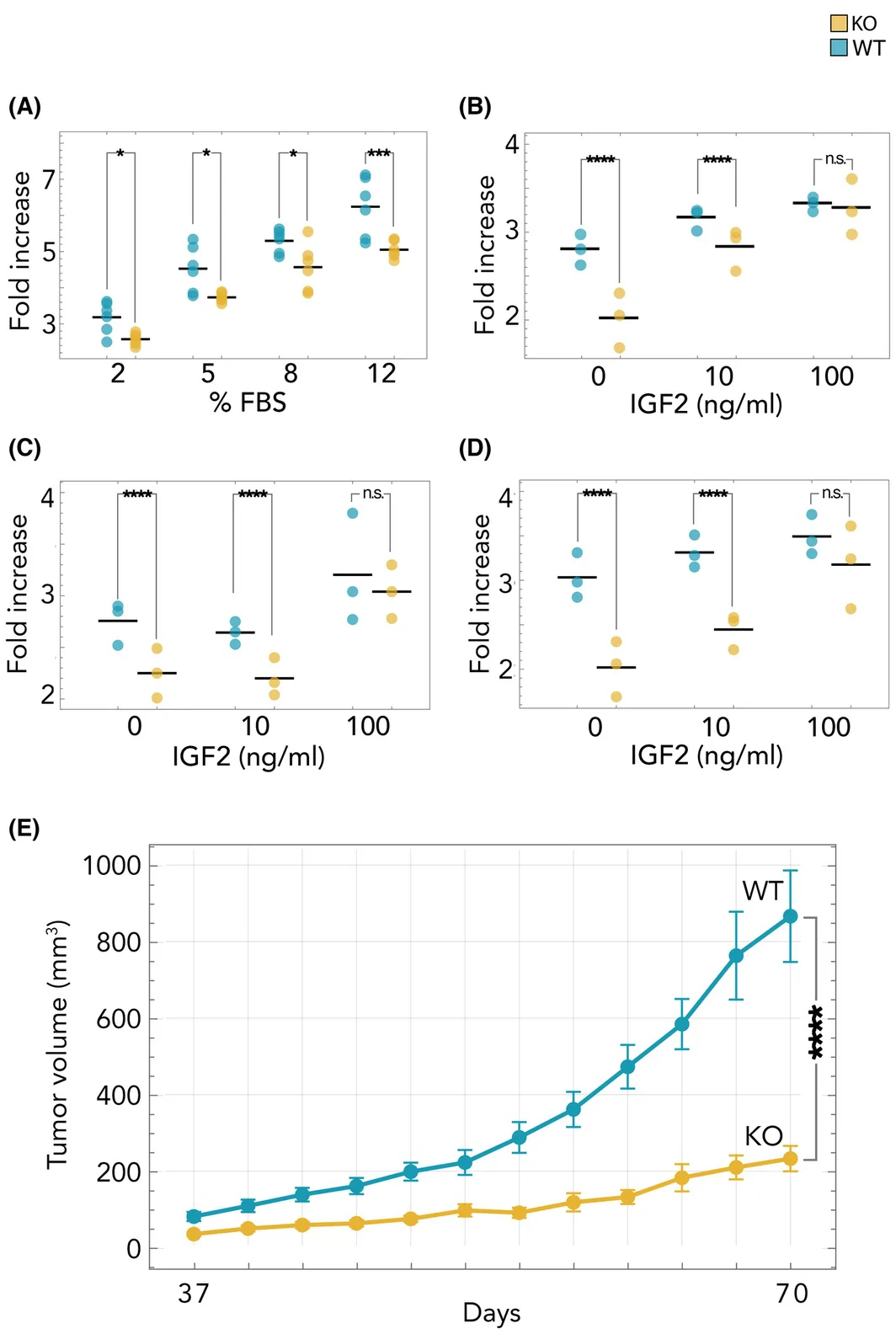
Proliferation of wild‐type and knockout Saos2 cells. Growth (fold increase) of WT (left, blue) and KO (right, yellow) cells after 6 days, starting from 6 × 10^4^ cells, under different amounts of (A) fetal bovine serum (FBS) or IGF2 in 4% FBS (B clone 1; C, clone 2; D clone 3). (E) Tumor volume over time in mouse xenografts (*n* = 6 per group) derived from WT or KO (clone 1) cells. The error bars show the standard error; * *P* < 0.05; ** *P* < 0.01; *** *P* < 0.005; **** *P* < 0.001 (Mann–Whitney U test).

*In vitro*, IGF2 KO cells exhibited reduced proliferation compared with wild‐type (WT) Saos2 cells across all fetal bovine serum (FBS) concentrations tested, although measurable growth persisted. Both WT and KO cells demonstrated enhanced proliferation in response to increasing FBS, indicating that serum factors generally promote growth; however, increased FBS did not compensate for the loss of IGF2 (Fig. 2A). In contrast, addition of exogenous IGF2 to the culture medium significantly enhanced proliferation of KO cells and substantially restored their growth to levels comparable to WT cells (Fig. 2B–D), demonstrating that IGF2 itself is sufficient to rescue the proliferation defect and that its signaling pathway remains intact.

*In vivo*, IGF2 KO xenografts grew substantially slower than WT tumors (Fig. 2E). At the experimental endpoint, KO tumors were smaller in volume, yet tumor formation occurred in all animals. After 70 days, KO tumors reached only approximately 25% of the size of WT tumors, confirming that IGF2 contributes to tumor growth but is not strictly essential.

### Dependency and expression analysis reveals compensatory survival pathways in Saos2 cells

**Fig. 3:**
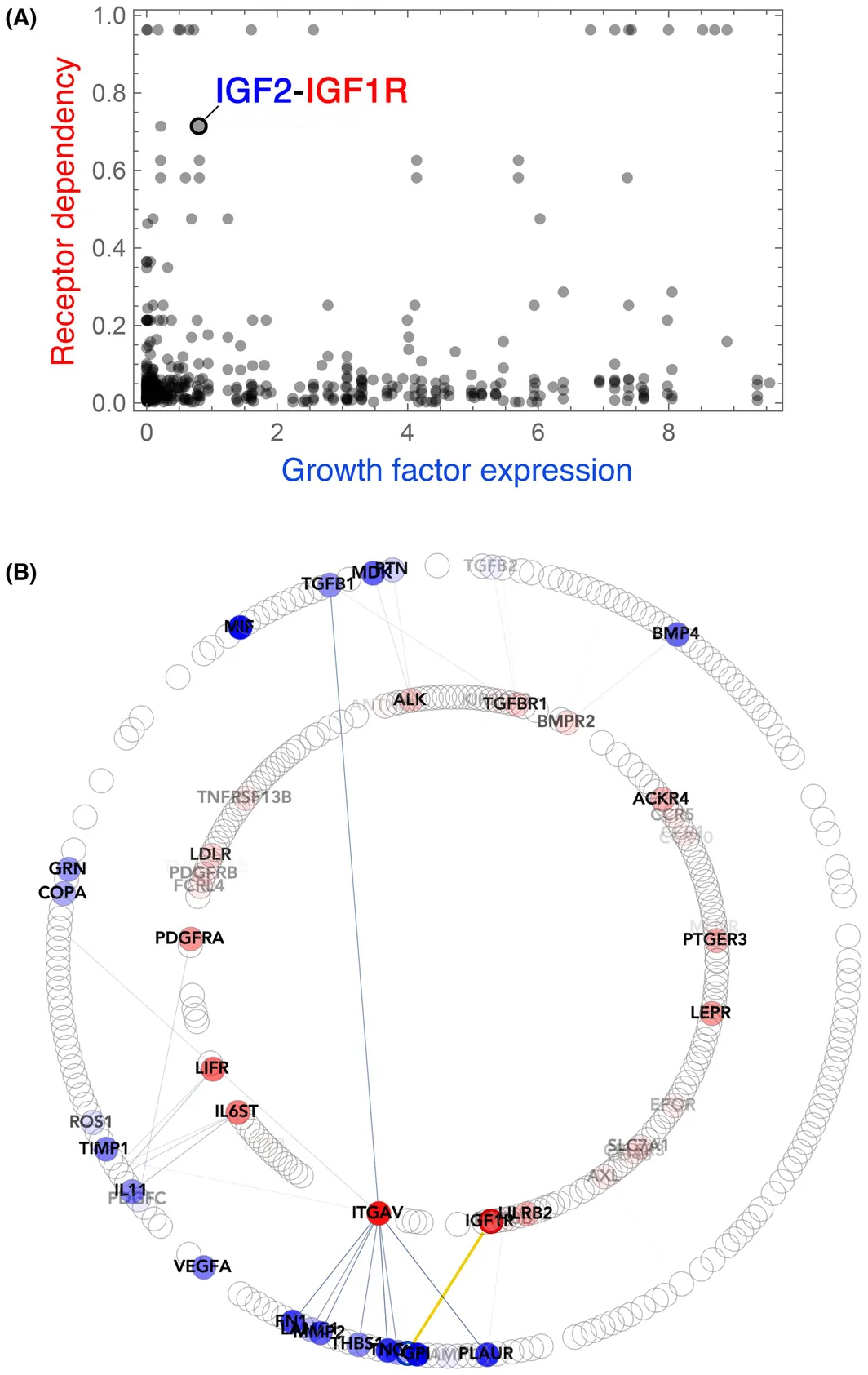
Growth factors and receptors in Saos2 cells. (A) Gene expression levels for growth factors, reported as log_2_ (TPM + 1); and gene dependency probabilities (higher values indicate greater likelihood that a given gene is essential in that cell line). Each dot represents a gene pair. IGF2‐IGF1R is highlighted. (B) The network of ligand–receptor pairs in Saos2. Lines join a ligand (external circle, blue) and a receptor (internal circle, red) pair: opacity is proportional to expression level (for ligands) or gene dependency (for receptors), and the opacity of the line connecting them is proportional to the importance of the pair, which is calculated by multiplying the expression level by the dependency score. The yellow line connects IGF2 with IGF1R. Network generated in Mathematica 14.1 (Wolfram, Inc.) using the values from Table 1.

To characterize the constitutive survival landscape of Saos2 cells, we compared gene expression data with DepMap dependency scores (Fig. 3A, Table 1) for known ligand–receptor pairs (Fig. 3B). While IGF1R showed a high dependency (0.71), IGF2 showed low expression (0.80), suggesting a potentially context‐dependent survival pathway or that minimal ligand input may be sufficient to sustain signaling.

**Table 1:** Expression level of growth factors and dependency of receptors in Saos2 cells. Gene expression levels for growth factors are reported as log_2_ (TPM + 1). Gene dependency is reported as the probability that a gene is essential. The growth factor–receptor pairs with the highest combined (product) of expression and dependency are listed.

Growth factor		Receptor	
Name	Expression	Name	Dependency
PLAUR	8.89	ITGAV	0.96
TNC	8.70	ITGAV	0.96
FN1	8.52	ITGAV	0.96
MMP2	8.00	ITGAV	0.96
THBS1	7.43	ITGAV	0.96
TGFB1	7.39	ITGAV	0.96
THY1	7.17	ITGAV	0.96
LAMC1	6.80	ITGAV	0.96
IL11	7.37	IL6ST	0.58
LIF	5.70	LIFR	0.63
LIF	5.70	IL6ST	0.58
PDGFC	6.03	PDGFRA	0.48
CLCF1	4.14	LIFR	0.63
FGF1	2.56	ITGAV	0.96
CLCF1	4.14	IL6ST	0.58
MDK	8.05	ALK	0.29
TGFB1	7.39	TGFBR1	0.25
PTN	6.38	ALK	0.29
BMP4	7.98	BMPR2	0.21
FGF2	1.60	ITGAV	0.96
TGFB2	5.94	TGFBR1	0.25
PLAUR	8.89	BMP8B	0.16
TGFB3	4.11	TGFBR1	0.25
GAS6	5.47	AXL	0.16
BMPR1A	3.99	BMPR2	0.21
GRN	7.17	TNFRSF1B	0.10
SPP1	0.72	ITGAV	0.96
MDK	8.05	LRP1	0.09
GDF11	2.78	TGFBR1	0.25
CSF1	4.02	SLC7A1	0.17
L1CAM	0.65	ITGAV	0.96
JAG1	4.73	CD46	0.13
PDGFB	1.24	PDGFRA	0.48
MIF	9.36	EGFR	0.06
IGF2	0.80	IGF1R	0.71

Analysis of other growth factor – receptor pairs suggests that several factors could partially substitute for IGF2 function in Saos2 cells, including ITGAV (integrin αV), LIFR (Leukemia Inhibitory Factor Receptor), IL6ST (Interleukin 6 Signal Transducer), and PDGFRA (Platelet‐Derived Growth Factor Receptor Alpha). ITGAV emerged as the most essential receptor (dependency score: 0.96), and multiple ligands capable of engaging ITGAV were highly expressed: PLAUR (expression: 8.89), TNC (8.70), FN1 (8.52), MMP2 (8.00), THBS1 (7.43), TGFB1 (7.39), THY1 (7.17), LAMC1 (6.80), FGF1 (2.56), FGF2 (1.60), and SPP1 (0.72). This suggests the presence of a robust autocrine and/or paracrine extracellular matrix (ECM) signaling network and that ITGAV‐mediated adhesion and ECM interactions are critical for cell survival and proliferation that may buffer the impact of IGF2 loss.

Analysis of cytokine signaling revealed that the LIF–LIFR and CLCF1–LIFR/IL6ST axes are moderately essential, with dependency scores of 0.63 for LIFR and 0.58 for IL6ST. LIF (expression 5.70) and CLCF1 (expression 4.14) are expressed at moderate levels, suggesting the existence of redundant or complementary autocrine loops supporting JAK/STAT‐mediated survival and proliferation. Similarly, PDGF signaling via PDGFC (expression 6.03) and PDGFB (expression 1.24) acting on PDGFRA (dependency 0.48) appears moderately essential, likely contributing to cell proliferation and motility.

Receptor tyrosine kinases, including ALK (engaged by MDK 8.05 and PTN 6.38; dependency 0.29) and AXL (GAS6 5.47; dependency 0.16), showed lower dependency, indicating supportive but noncritical roles. TGFβ and BMP pathways, involving TGFB1–3 and BMP4/BMPR1A signaling through TGFBR1 (0.25) and BMPR2 (0.21), were also lowly essential, suggesting potential modulatory functions rather than critical roles in basal survival.

Overall integration of ligand expression and receptor dependency predicts several autocrine loops: a dominant essential pathway driven by ITGAV–ECM ligands; LIF/CLCF1 binding to LIFR/IL6ST, likely supporting JAK/STAT survival signaling; PDGFC/PDGFB binding to PDGFRA, likely contributing to proliferation and motility. IGF2 binding to IGF1R. Other ligands, including GAS6–AXL and BMPs, likely play secondary, supportive roles under basal conditions. These results indicate that the cell line relies, at least *in vitro*, predominantly on ITGAV‐mediated integrin signaling for survival and proliferation, with moderately essential cytokine and PDGF pathways providing supportive growth signals. RTK and TGFβ/BMP pathways appear nonessential under basal conditions but may contribute under stress or *in vivo*.

### Differential gene expression analysis reveals limited transcriptional changes following IGF2 loss

Finally, gene set enrichment analysis (GSEA) was performed using a ranked list of all genes ordered by log_2_ fold change between WT and IGF2‐knockout cells. Enrichment was evaluated across the full transcriptome without imposing fold change thresholds. This ranked‐list framework assesses whether members of predefined gene sets accumulate preferentially at the extremes of the ranked distribution, rather than relying on arbitrarily thresholded gene lists [36, 37]. Negative normalized enrichment scores (NES) indicate relative depletion of a gene set in KO cells, whereas positive NES indicate relative enrichment. Significance estimates were derived from the GSEA permutation framework and are reported as FDR‐adjusted *P*‐values.

**Fig. 4:**
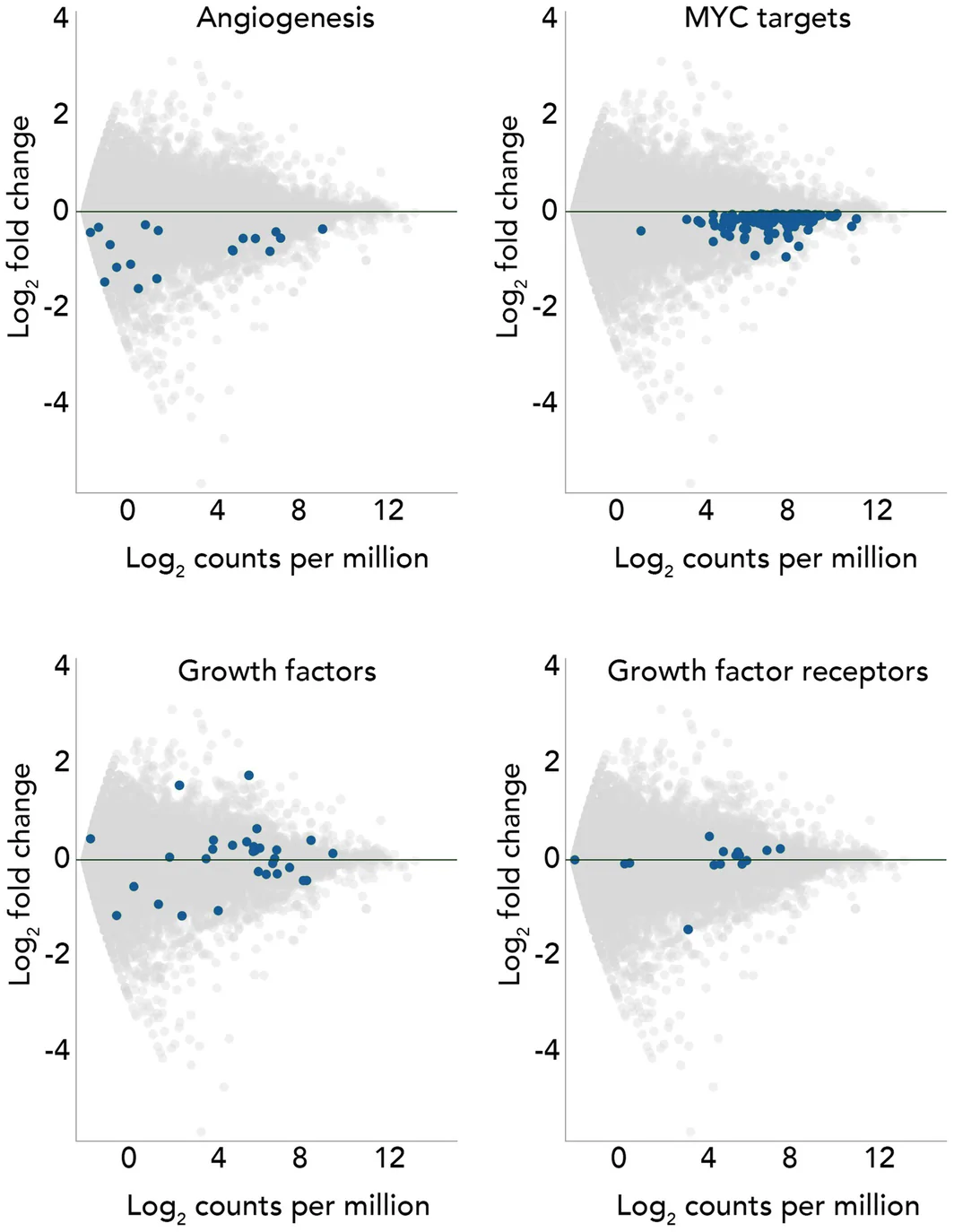
Differential gene expression in wild‐type and knockout Saos2 cells. Each point (gray) represents a single gene, with the x‐axis showing the average expression level (log_2_ counts per million) and the y‐axis showing the expression change (log_2_ fold change). Positive values indicate higher expression in KO cells, and negative values indicate higher expression in WT cells. The horizontal line at log_2_ fold change = 0 indicates no expression change. Highlighted (dark blue) points represent genes involved in angiogenesis, MYC Targets gene sets, or the growth factors and their receptors listed in Table 1.

Of the Hallmark gene sets evaluated, we focus on those showing statistical significance (Data S2) and relevance to the phenotypes described above. Ranked GSEA (Fig. 4) revealed coordinated depletion of angiogenesis‐related gene sets in IGF2 KO cells (NES = −1.579; FDR = 0.041), suggesting reduced representation of angiogenic programs across the transcriptome. The MYC Targets V1 and V2 gene sets showed similar negative enrichment trends, although these did not reach statistical significance. No enrichment changes were observed in gene sets directly associated with canonical IGF‐axis signaling. Likewise, no enrichment of ITGAV‐related gene sets was detected, indicating that the ITGAV‐mediated interactions described above likely represent a pre‐existing survival pathway rather than a transcriptional consequence of IGF2 loss.

Collectively, these results indicate that, while IGF2 contributes to the proliferation of Saos2 cells *in vitro*, and significantly enhances the growth of Saos2 xenografts, its loss can be tolerated, and compensatory growth does not appear to be mediated by major changes in canonical IGF2 pathway genes but may be supported by constitutively active ITGAV–ECM signaling, as suggested by the integration of expression and dependency data.

## Discussion

In this study, we investigated the functional contribution of endogenous IGF2 to osteosarcoma growth using a CRISPR–Cas9 IGF2 knockout model in Saos2 cells [31, 32]. Our results show that loss of IGF2 reduces, but does not abolish, *in vitro* proliferation, and leads to markedly smaller tumors *in vivo*. These results indicate that IGF2 functions primarily as a growth enhancer rather than an essential survival factor in OS, consistent with its known activation of IGF1R and downstream PI3K/AKT and MAPK signaling pathways [4, 5, 6, 7, 8, 9, 10, 11, 12, 13, 14, 15].

Across multiple serum conditions, IGF2 KO cells proliferated more slowly than wild‐type (WT) cells but retained measurable growth. Increasing FBS concentrations enhanced proliferation in both genotypes; however, high serum did not compensate for the loss of IGF2, indicating that serum‐derived mitogens cannot fully substitute for endogenous ligand. Exogenous IGF2 restored KO proliferation to WT levels, confirming that the IGF signaling machinery remains intact and that the primary defect arises from ligand absence rather than altered receptor expression or downstream signaling competence. This observation also supports the concept that paracrine IGF2 from neighboring tumor or stromal cells could partially rescue proliferation *in vivo*, as observed in other systems such as liver cells and glioma lines [26, 27, 28, 29, 30, 43].

The *in vivo* xenograft data mirrored these trends but revealed a stronger effect. IGF2 KO tumors formed in all mice, confirming that IGF2 is not required for tumor initiation. However, KO tumors grew substantially slower and reached only ~25% of WT tumor size at the endpoint, indicating that endogenous IGF2 significantly contributes to tumor expansion. To elucidate the role of IGF2 in initial tumor formation, limiting dilution experiments should be performed. It should be noted that, since human IGF2 can stimulate mouse cells [44], murine IGF2 in the xenograft microenvironment may partially stimulate human IGF1R signaling in Saos2 cells and contribute to formation and residual tumor growth *in vivo*.

Although IGF2 is known to activate IGF1R and initiate receptor tyrosine kinase signaling cascades—including PI3K–AKT–mTOR, RAS–RAF–MEK–ERK, and JAK/STAT [4, 5, 6, 7, 8, 9, 10, 11, 12, 13, 14, 15]—direct measurement of pathway activation was not performed here and should be pursued in future work. Nevertheless, the observed slower proliferation in KO cells is consistent with reduced signaling through these pathways, while the retention of measurable growth suggests that compensatory mechanisms partially mitigate the loss of IGF2. It is worth noting that IGF1R is a receptor for both IGF1 and IGF2, and that IGF1R dependency does not specify ligand dominance. However, the strong proliferation defect following IGF2 deletion demonstrates that IGF2 contributes nonredundantly to IGF1R signaling.

The relatively low expression of IGF2 (0.80, CCLE log‐scale) may appear inconsistent with the substantial phenotypic effect of its knockout. Several considerations may reconcile this apparent discrepancy. First, CCLE expression values are derived from standard *in vitro* culture conditions and may not reflect IGF2 levels *in vivo*, where microenvironmental cues such as hypoxia or nutrient stress could upregulate its expression. Second, even low levels of autocrine IGF2 may be sufficient to sustain IGF1R activation, particularly given the high dependency score of IGF1R (0.71) in this cell line—a receptor primed for signaling may require only minimal ligand input. Third, IGF1R can also be activated by IGF1 and, at lower affinity, by insulin, raising the possibility that multiple ligands converge on this receptor, with IGF2 providing a nonredundant contribution that becomes apparent only upon its complete removal.

Integration with receptor dependency data provides further insight. While IGF1R dependency is moderate and expression of IGF2 is low, Saos2 cells show very high dependency on ITGAV, consistent with strong expression of multiple ITGAV ligands, including PLAUR, TNC, FN1, MMP2, and THBS1. This dominant integrin–ECM axis likely sustains basal survival [45, 46] and explains why IGF2‐KO cells retain measurable growth *in vitro*. Additional moderate contributors, such as LIFR/IL6ST and PDGFRA, may further support proliferation via partially overlapping pathways [47, 48], but none fully replace IGF2 signaling. This contextual analysis helps explain how IGF2 deletion reduces proliferation without abolishing viability.

Further insight from transcriptomic analysis revealed negative enrichment of angiogenesis‐related gene sets in IGF2 KO cells, suggesting that IGF2 may promote tumor growth not only through intrinsic proliferation but also by supporting vascular‐associated programs. This provides a potential explanation for why the *in vivo* effect (75% tumor reduction) exceeded the *in vitro* proliferation deficit: reduced angiogenic signaling would preferentially impair tumor expansion *in vivo*, where vascular support is required, but not *in vitro*, where nutrients are supplied exogenously. Reduced MYC target gene expression is consistent with diminished proliferative drive downstream of IGF signaling.

No enrichment changes were observed in canonical IGF‐axis gene sets, supporting the interpretation that the KO phenotype reflects loss of ligand input rather than broad transcriptional reprogramming of the IGF signaling network.

Our study has some limitations. First, experiments were performed in a single, albeit well‐established, OS cell line, and IGF2 dependence may vary across subtypes or patient‐derived models. While our rescue experiment mitigates this concern (since exogenous IGF2 restores growth, the phenotype is IGF2‐dependent, not clone‐dependent), the result cannot be generalized to all osteosarcomas. Second, transcriptomic analyses were limited to mRNA expression; protein levels, receptor phosphorylation, secretion, or stromal interactions were not evaluated and represent priorities for future work. Third, although angiogenesis gene sets were altered, we did not directly assess tumor vascularization or perfusion. Fourth, IGF2 re‐expression experiments were not conducted, though complete rescue by exogenous IGF2 mitigates concerns about specificity. Finally, manual hemocytometer counting is subject to sampling variability and observer bias, particularly at high cell densities; automated imaging‐based counters could reduce this in future work.

Even with these limitations, our results suggest that endogenous IGF2 supports both *in vitro* proliferation and *in vivo* tumor expansion in osteosarcoma but is not strictly essential for survival or tumor initiation. The dominant ITGAV–ECM axis, together with moderate contributions from LIFR, IL6ST, and PDGFRA, likely sustains basal proliferation in the absence of IGF2. Future studies examining receptor activation, angiogenesis, and stromal contributions will be important for fully defining the role of IGF2 and the broader IGF axis in osteosarcoma progression.

## Conflict of interest

The authors declare no conflict of interest.

## Author contributions

SY and MA conceived and designed the project. SY performed the experiments. MA performed the bioinformatics analysis. SY and MA interpreted the results. MA wrote the paper.

## Supporting information


**Data S1.** RNA‐seq differential gene expression.

**Data S2.** RNA‐seq gene set enrichment analysis.

**Data S3.** RNA‐seq gene biotype categories.

**Data S4.** RNA‐seq alignment and mapping QC metrics for the KO cell line (clone 1).

**Data S5.** RNA‐seq alignment and mapping QC metrics for the WT cell line.

## Data Availability

The data that support the findings of this study are available in the supporting information of this article.

## References

[1] Beird HC , Bielack SS , Flanagan AM , Gill J , Heymann D , Janeway KA , Livingston JA , Roberts RD , Strauss SJ and Gorlick R (2022) Osteosarcoma. Nat Rev Dis Primers 8, 77. 36481668 10.1038/s41572-022-00409-y

[2] Whelan JS and Davis LE (2018) Osteosarcoma, Chondrosarcoma, and Chordoma. J Clin Oncol 36, 188–193. 29220289 10.1200/JCO.2017.75.1743

[3] Isakoff MS , Bielack SS , Meltzer P and Gorlick R (2015) Osteosarcoma: current treatment and a collaborative pathway to success. J Clin Oncol 33, 3029–3035. 26304877 10.1200/JCO.2014.59.4895 PMC4979196

[4] Pollak M (2008) Insulin and insulin‐like growth factor signalling in neoplasia. Nat Rev Cancer 8, 915–928. 19029956 10.1038/nrc2536

[5] Samani AA , Yakar S , LeRoith D and Brodt P (2007) The role of the IGF system in cancer growth and metastasis: overview and recent insights. Endocr Rev 28, 20–47. 16931767 10.1210/er.2006-0001

[6] LeRoith D and Roberts CT (2003) The insulin‐like growth factor system and cancer. Cancer Lett 195, 127–137. 12767520 10.1016/s0304-3835(03)00159-9

[7] Belfiore A and Malaguarnera R (2011) Insulin receptor and cancer. Endocr Relat Cancer 18, R125–R147. 21606157 10.1530/ERC-11-0074

[8] Livingstone C (2013) IGF2 and cancer. Endocr Relat Cancer 20, R321–R339. 24080445 10.1530/ERC-13-0231

[9] Yu H and Rohan T (2000) Role of the insulin‐like growth factor family in cancer development and progression. J Natl Cancer Inst 92, 1472–1489. 10995803 10.1093/jnci/92.18.1472

[10] Morrione A , Valentinis B , Xu SQ , Yumet G , Louvi A , Efstratiadis A and Baserga R (1997) Insulin‐like growth factor II stimulates cell proliferation through the insulin receptor. Proc Natl Acad Sci USA 94, 3777–3782. 9108054 10.1073/pnas.94.8.3777 PMC20517

[11] Siddle K (2011) Signalling by insulin and IGF receptors: supporting acts and new players. J Mol Endocrinol 47, R1–R10. 21498522 10.1530/JME-11-0022

[12] Li J , Choi E , Yu H and Bai XC (2019) Structural basis of the activation of type 1 insulin‐like growth factor receptor. Nat Commun 10, 4567. 31594955 10.1038/s41467-019-12564-0 PMC6783537

[13] Manning BD and Cantley LC (2007) AKT/PKB signaling: navigating downstream. Cell 129, 1261–1274. 17604717 10.1016/j.cell.2007.06.009 PMC2756685

[14] Mendoza MC , Er EE and Blenis J (2011) The Ras‐ERK and PI3K‐mTOR pathways: cross‐talk and compensation. Trends Biochem Sci 36, 320–328. 21531565 10.1016/j.tibs.2011.03.006 PMC3112285

[15] Zoncu R , Efeyan A and Sabatini DM (2011) mTOR: from growth signal integration to cancer, diabetes and ageing. Nat Rev Mol Cell Biol 12, 21–35. 21157483 10.1038/nrm3025 PMC3390257

[16] Scotlandi K , Manara MC and Serra M (2009) Insulin‐like growth factor system in osteosarcoma. Cancer Treat Res 152, 81–96.

[17] Behjati S , Tarpey PS , Haase K , Ye H , Young MD , Alexandrov LB , Farndon SJ , Collord G , Wedge DC , Martincorena I *et al*. (2017) Recurrent mutation of IGF signalling genes and distinct patterns of genomic rearrangement in osteosarcoma. Nat Commun 8, 15936. 28643781 10.1038/ncomms15936 PMC5490007

[18] Taylor AM , Sun JM , Yu A , Voicu H , Shen J , Barkauskas DA , Triche TJ , Gastier‐Foster JM , Man TK and Lau CC (2022) Integrated DNA copy number and expression profiling identifies IGF1R as a prognostic biomarker in pediatric osteosarcoma. Int J Mol Sci 23, 8036. 35887382 10.3390/ijms23148036 PMC9319262

[19] Wang YH , Han XD , Qiu Y , Xiong J , Yu Y , Wang B , Zhu ZZ , Qian BP , Chen YX , Wang SF *et al*. (2012) Increased expression of insulin‐like growth factor‐1 receptor is correlated with tumor metastasis and prognosis in patients with osteosarcoma. J Surg Oncol 105, 235–243. 21866554 10.1002/jso.22077

[20] Shimizu T , Sugihara E , Yamaguchi‐Iwai S , Tamaki S , Koyama Y , Kamel W , Ueki A , Ishikawa T , Chiyoda T , Osuka S *et al*. (2014) IGF2 preserves osteosarcoma cell survival by creating an autophagic state of dormancy that protects cells against chemotherapeutic stress. Cancer Res 74, 6531–6541. 25273088 10.1158/0008-5472.CAN-14-0914

[21] Haisa M (2013) The type 1 insulin‐like growth factor receptor signalling system and targeted tyrosine kinase inhibition in cancer. J Int Med Res 41, 253–264. 23569026 10.1177/0300060513476585

[22] Ameline B , Kovac M , Nathrath M , Barenboim M , Witt O , Krieg AH and Baumhoer D (2021) Overactivation of the IGF signalling pathway in osteosarcoma: a potential therapeutic target? J Pathol Clin Res 7, 165–172. 33295144 10.1002/cjp2.191 PMC7869926

[23] Kuijjer ML , Peterse EF , van den Akker BE , Briaire‐de Bruijn IH , Serra M , Meza‐Zepeda LA , Myklebost O , Hassan AB , Hogendoorn PC and Cleton‐Jansen AM (2013) IR/IGF1R signaling as potential target for treatment of high‐grade osteosarcoma. BMC Cancer 13, 245. 23688189 10.1186/1471-2407-13-245 PMC3672007

[24] Olmos D , Tan DS , Jones RL and Judson IR (2010) Biological rationale and clinical development of IGF1R inhibitors in sarcomas. Ann Oncol 21, 1097–1108.

[25] Pappo AS , Patel SR , Crowley J , Reinke DK , Kuenkele KP , Chawla SP , Toner GC , Maki RG , Meyers PA , Chugh R *et al*. (2011) R1507, a monoclonal antibody to the insulin‐like growth factor 1 receptor, in patients with recurrent or refractory Ewing sarcoma family of tumors and osteosarcoma. J Clin Oncol 29, 4541–4547. 22025149 10.1200/JCO.2010.34.0000 PMC3236654

[26] Zhang M , Xuan S , Bouxsein ML , von Stechow D , Akeno N , Faugere MC , Malluche H , Zhao G , Rosen CJ , Efstratiadis A *et al*. (2002) Osteoblast‐specific knockout of the insulin‐like growth factor receptor gene reveals an essential role of IGF signaling in bone matrix mineralization. J Biol Chem 277, 44005–44012. 12215457 10.1074/jbc.M208265200

[27] Rogers MA , Kalter V , Strowitzki M , Schneider M and Lichter P (2016) IGF2 knockdown in two colorectal cancer cell lines decreases survival, adhesion and modulates survival‐associated genes. Tumour Biol 37, 12485–12495. 27337954 10.1007/s13277-016-5115-x

[28] Gui W , Zhu Y , Sun S , Zhu W , Tan B , Zhao H , Shang C , Zheng F , Lin X and Li H (2021) Knockdown of insulin‐like growth factor 2 gene disrupts mitochondrial functions in the liver. J Mol Cell Biol 13, 543–555. 33988719 10.1093/jmcb/mjab030 PMC8697341

[29] Sun H , Zhou L , Zhang Y , Guo S , Pan X and Li C (2020) IGF2 secreted by glioma‐associated mesenchymal stem cells promotes glioma cell proliferation through IGF1R signaling. J Exp Clin Cancer Res 39, 82.32381104

[30] Liu J , Hu X , Chen J , Li X , Wang L , Wang B , Peng W , Yang C , Li Z , Chen Y *et al*. (2017) Pericentral hepatocytes produce insulin‐like growth factor‐2 to promote liver regeneration during selected injuries in mice. Hepatology 66, 2002–2015. 28653763 10.1002/hep.29340

[31] Tsherniak A , Vazquez F , Montgomery PG , Weir BA , Kryukov G , Cowley GS , Gill S , Harrington WF , Pantel S , Krill‐Burger JM *et al*. (2017) Defining a cancer dependency map. Cell 170, 564–576. 28753430 10.1016/j.cell.2017.06.010 PMC5667678

[32] Hart T , Chandrashekhar M , Aregger M , Steinhart Z , Brown KR , MacLeod G , Mis M , Zimmermann M , Fradet‐Turcotte A , Sun S *et al*. (2015) High‐resolution CRISPR screens reveal fitness genes and genotype‐specific cancer liabilities. Cell 163, 1515–1526. 26627737 10.1016/j.cell.2015.11.015

[33] Rodan SB , Imai Y , Thiede MA , Wesolowski G , Thompson D , Bar‐Shavit Z , Shull S , Mann K and Rodan GA (1987) Characterization of a human osteosarcoma cell line (Saos‐2) with osteoblastic properties. Cancer Res 47, 4961–4966. 3040234

[34] Labun K , Montague TG , Krause M , Torres Cleuren YN , Tjeldnes H and Valen E (2019) CHOPCHOP v3: expanding the CRISPR web toolbox beyond genome editing. Nucleic Acids Res 47, W171–W174. 31106371 10.1093/nar/gkz365 PMC6602426

[35] Efremova M , Vento‐Tormo M , Teichmann SA and Vento‐Tormo R (2020) CellPhoneDB: inferring cell–cell communication from combined expression of multi‐subunit ligand–receptor complexes. Nat Protoc 15, 1484–1506. 32103204 10.1038/s41596-020-0292-x

[36] Barbie DA , Tamayo P , Boehm JS , Kim SY , Moody SE , Dunn IF , Schinzel AC , Sandy P , Meylan E , Scholl C *et al*. (2009) Systematic RNA interference reveals that oncogenic KRAS‐driven cancers require TBK1. Nature 462, 108–112. 19847166 10.1038/nature08460 PMC2783335

[37] Subramanian A , Tamayo P , Mootha VK , Mukherjee S , Ebert BL , Gillette MA , Paulovich A , Pomeroy SL , Golub TR , Lander ES *et al*. (2005) Gene set enrichment analysis: a knowledge‐based approach for interpreting genome‐wide expression profiles. Proc Natl Acad Sci USA 102, 15545–15550. 16199517 10.1073/pnas.0506580102 PMC1239896

[38] Fang Z , Liu X and Peltz G (2023) GSEApy: a comprehensive package for performing gene set enrichment analysis in python. Bioinformatics 39, btac757. 36426870 10.1093/bioinformatics/btac757 PMC9805564

[39] Liberzon A , Subramanian A , Pinchback R , Thorvaldsdóttir H , Tamayo P and Mesirov JP (2011) Molecular signatures database (MSigDB) 3.0. Bioinformatics 27, 1739–1740. 21546393 10.1093/bioinformatics/btr260 PMC3106198

[40] Liberzon A , Birger C , Thorvaldsdóttir H , Ghandi M , Mesirov JP and Tamayo P (2015) The molecular signatures database Hallmark gene set collection. Cell Systems 1, 417–425. 26771021 10.1016/j.cels.2015.12.004 PMC4707969

[41] Barretina J , Caponigro G , Stransky N , Venkatesan K , Margolin AA , Kim S , Wilson CJ , Lehár J , Kryukov GV , Sonkin D *et al*. (2012) The cancer cell line encyclopedia enables predictive modelling of anticancer drug sensitivity. Nature 483, 603–607. 22460905 10.1038/nature11003 PMC3320027

[42] Ghandi M , Huang FW , Jané‐Valbuena J , Kryukov GV , Lo CC , McDonald ER , Barretina J , Gelfand ET , Bielski CM , Li H *et al*. (2019) Next‐generation characterization of the cancer cell line encyclopedia. Nature 569, 503–508. 31068700 10.1038/s41586-019-1186-3 PMC6697103

[43] Unger C , Kramer N , Unterleuthner D , Scherzer M , Burian A , Rudisch A , Stadler M , Schlederer M , Lenhardt D , Riedl A *et al*. (2017) Stromal‐derived IGF2 promotes colon cancer progression via paracrine and autocrine mechanisms. Oncogene 36, 5341–5355. 28534511 10.1038/onc.2017.116

[44] Muhammad T , Wan Y , Sha Q , Wang J , Huang T , Cao Y , Li M , Yu X , Yin Y , Chan WY *et al*. (2020) IGF2 improves the developmental competency and meiotic structure of oocytes from aged mice. Aging (Albany NY) 13, 2118–2134. 33318299 10.18632/aging.202214 PMC7880328

[45] Desgrosellier JS and Cheresh DA (2010) Integrins in cancer: biological implications and therapeutic opportunities. Nat Rev Cancer 10, 9–22. 20029421 10.1038/nrc2748 PMC4383089

[46] Desgrosellier JS , Barnes LA , Shields DJ , Huang M , Lau SK , Prévost N , Tarin D , Shattil SJ and Cheresh DA (2009) Integrin αvβ3/c‐src oncogenic unit promotes Anchorage‐independence and tumor progression. Nat Med 15, 1163–1169. 19734908 10.1038/nm.2009 PMC2759406

[47] Andrae J , Gallini R and Betsholtz C (2008) Role of platelet‐derived growth factors in physiology and medicine. Genes Dev 22, 1276–1312. 18483217 10.1101/gad.1653708 PMC2732412

[48] Tallquist MD and Kazlauskas A (2004) PDGF signaling in cells and mice. Cytokine Growth Factor Rev 15, 205–213. 15207812 10.1016/j.cytogfr.2004.03.003

